# Promising New Treatments for Psoriasis

**DOI:** 10.1155/2013/980419

**Published:** 2013-07-01

**Authors:** Sarah Dubois Declercq, Roxane Pouliot

**Affiliations:** ^1^Centre LOEX de l'Université Laval, Génie Tissulaire et Régénération, LOEX-Centre de Recherche FRSQ du CHU, Aile-R, 1401 18e rue, Québec, QC, Canada G1J 1Z4; ^2^Faculté de Pharmacie, Université Laval, Québec, QC, Canada G1V 0A6

## Abstract

Psoriasis is a chronic, proliferative, and inflammatory skin disease affecting 2-3% of the population and is characterized by red plaques with white scales. Psoriasis is a disease that can affect many aspects of professional and social life. Currently, several treatments are available to help control psoriasis such as methotrexate, ciclosporin, and oral retinoids. However, the available treatments are only able to relieve the symptoms and lives of individuals. The discovery of new immunological factors and a better understanding of psoriasis have turned to the use of immunological pathways and could develop new biological drugs against specific immunological elements that cause psoriasis. Biological drugs are less toxic to the body and more effective than traditional therapies. Thus, they should improve the quality of life of patients with psoriasis. This review describes new psoriasis treatments, which are on the market or currently in clinical trials that are being used to treat moderate-to-severe plaque psoriasis. In addition, this paper describes the characteristics and mechanisms in detail. In general, biological drugs are well tolerated and appear to be an effective alternative to conventional therapies. However, their effectiveness and long-term side effects need to be further researched.

## 1. Introduction

The objective of this paper is to present a literature review of the various psoriatic treatments currently available on the market. This review describes the mechanisms and the characteristics of the most widely used psoriatic treatments.

Psoriasis is a noncontagious chronic inflammatory dermatosis affecting 2% of the world population [1]. Psoriasis is characterized by recurrent episodes of red and scaly skin plaques that are sharply demarcated from adjacent normal skin [2]. This disease is partly due to a genetic predisposition and other environmental factors [3]. Psoriasis is a serious skin disease that affects a person's daily life on many levels including professional and social life. The physical and psychological impacts of psoriasis are comparable to those of cancer, heart disease, diabetes, or depression [4]. The percentage of the body affected by psoriatic plaques can vary. It is possible to observe mild (<2%), moderate (2–10%), and severe (>10%) psoriasis in different people [5]. The most common type of psoriasis is chronic plaque psoriasis or psoriasis vulgaris [6]. However, the disease can also be classified into 4 different types such as guttate, pustular, erythrodermic, and inverse psoriasis [7].

The histological characteristics are the following: epidermal hyperplasia (abnormal differentiation and incomplete maturation of keratinocytes), a thickened epidermis, and a reduced or absent granular layer. This is caused by hyperproliferation and differentiation of fast epidermal keratinocytes which takes 7 to 10 days as opposed to 28–50 days for healthy skin. Epidermal infiltration of immune cells (T cells) [8] and CD11c^+^ dendritic cells in the dermis can be observed. CD8^+^ cells and neutrophils are found in the epidermis [9]. In addition to these anomalies, an increase in the formation process of new blood vessels (angiogenesis) and inflammation of the skin [10] can be observed. 

Psoriasis has been long thought to be caused by hyperproliferation of keratinocytes. However, when immunomodulatory treatments became effective, the immune system was found to be an important factor in the development of the disease.

Psoriasis is a chronic inflammatory disease in which dendritic cells, T lymphocytes, macrophages, neutrophils, and keratinocytes are responsible for the initiation of skin lesions. Presentation of antigen and the formation of the immunological synapse will cause the secretion of various cytokines/chemokines and allow the differentiation of T cells into effector cells such as Th1, Th2, and Th17. Thus, each effector cell will secrete particular cytokines. It has been shown that IFN-*α*, TNF-*α*, and IL-2 increased the proliferation of keratinocytes [11]. TNF-*α* activates the development of lesions by increasing the number of molecules involved in the inflammatory response or the adhesion molecules. IL-2 is an important stimulator of T cells but it does not have the ability to alter the production of cytokines or chemokines from healthy or psoriatic keratinocytes [12]. Activated keratinocytes produce cytokines and chemokines that bring lymphocytes to the site of inflammation and that deregulate their proliferation. Thus, subpopulations of Th1 and Th17 were found in psoriatic skin lesions [13]. Recent data from inflammatory skin models suggest that IL-23 (a key cytokine that has been found to play a critical role in the pathogenesis of psoriasis) and Th17 T cells (which produce IL-17 and IL-22) could be pivotal inducers of epidermal hyperplasia and thus modify epidermal differentiation in psoriasis [14].

The high production of vascular endothelial growth factors (VEGF) in psoriatic keratinocytes promotes angiogenesis, thereby causing increased vascularization and inflammation. Neutrophils are found in large quantities in psoriatic lesions. Thus, it has been shown that some cytokines such as IL-8 cause the accumulation of neutrophils in the skin [15].

Although many studies have been done on the possible causes of psoriasis, the origin of the disease remains unknown.

Currently, several treatments are available to help control psoriasis; however, the available treatments are only able to relieve the symptoms and lives of individuals [16]. The choice of the most appropriate treatment depends on the patient's general health, age, comorbidities, form and severity of the pathology, and, also, on the affected body parts [16].

**Figure 1:**
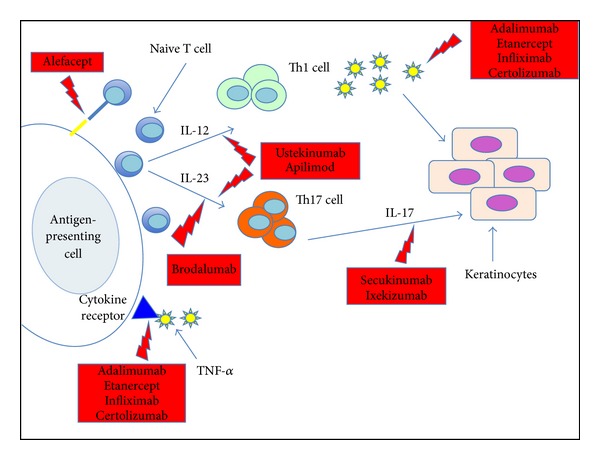
Schematic representation of biological therapy of psoriasis.

In recent years, new findings on the immunologic factors related to the disease have fundamentally changed the treatment of psoriasis and created new drugs. New psoriasis treatments are derived from biotechnologies and are called biological drugs (Figure 1, Table 1).

These new classes of treatments consist in the fusion of proteins and monoclonal antibodies that specifically target the activity of  T cells or inflammatory cytokines by inhibiting or modulating specific immune system actors. Biological drugs can save other organs and minimize side effects. These treatments are used for severe cases and are used as a last resort because they have a high cost and significant side effects. Nevertheless, biological therapy was associated with lower toxicity than the systemic treatments previously used [17]. Development of new biologics is favored because traditional topical therapies, phototherapy, and systemic medications have been associated with patient frustration [18]. Although biologics are more expensive than other forms of therapy, they may indirectly lessen costs for some patients by reducing the need or length of hospitalization [19]. Psoriatic patients on biologics show greater improvement than do patients on topicals, phototherapy, or conventional systemic agents, and both patients and their dermatologists express greater satisfaction with these biological therapies [20].

**Figure 2:**
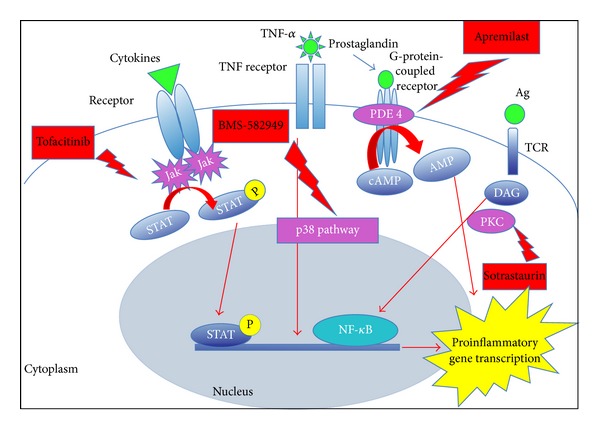
Schematic representation of the mechanism of action of the small-molecule inhibitors. Jak: janus kinase; STAT: signal transducing proteins and activator of transcription; STAT-P: phosphorylated STAT (active); PDE 4: phosphodiesterase 4; NF-KB: nuclear factor kappaB; PKC: protein kinases C; DAG: diacylglycerol; cAMP: cyclic adenosine monophosphate; AMP: adenosine monophosphate.

Research continues to elucidate new pathological mechanisms and develop new oral agents including Janus kinase (Jak), protein kinase C (PKC), and mitogen-activated protein kinase (MAPK) inhibitors (Figure 2). These proteins participate in biological processes involved in the immune response to psoriasis and are found in all cells.

Biological drugs can be classified into two categories according to their mechanisms. The two main classes that are currently available are the drugs that prevent the activation of T cells and those that target cytokines [21].

## 2. Anti-TNF-***α*** Treatments

It is known from the literature that sera of patients with psoriasis contain a high amount of tumor necrosis factor *α* (TNF-*α*) [2]. This cytokine is extremely proinflammatory and is very important in the development of inflammation in psoriasis. Indeed, TNF-*α* stimulates the production of cytokines and the adhesion of molecules by keratinocytes and thereby increases the recruitment of immune cells [22]. Anti-TNF-*α* has been developed to capture the TNF-*α* and to block its activity and consequently reduce the interactions between immune cells and keratinocytes. There are different molecules inhibiting TNF-*α* in the treatment of psoriasis. Currently, there are three TNF-*α* inhibitors that are approved for treatment [2]. The neutralization of the TNF-*α* prevents its interaction with receptors TNFR1. The binding of the TNF-*α* at the receptor level results in a cascade of pathways. This process is then used to activate the NF-KB1 which is a transcription factor that induces proliferation, cell survival, and cytokine production [23].

Infliximab is a chimeric monoclonal antibody that neutralizes TNF-*α* [24, 25]. This process causes the TNF-*α* to bind to the antibody and to deactivate it permanently. This treatment helps to inhibit the production of inflammatory cytokines.

Etanercept is a recombinant human TNF-*α* receptor fusion protein that neutralizes soluble TNF-*α* and which is administered subcutaneously twice per week. This synthetic receptor has a higher affinity for TNF-*α* than the natural receptor [26]. This treatment reduces psoriatic inflammation.

Adalimumab is a completely humanized monoclonal antibody IgG1 and is produced to capture the TNF-*α*. Adalimumab is administered subcutaneously every two weeks [27].

Certolizumab Pegol (Cimzia) is a recombinant, humanized anti-TNF-*α* antibody that is already on the market with indications for RA and Crohn's disease and has been studied for psoriasis (phase II results are available) [28].

## 3. Other Anticytokines Treatments

It is known from the literature that Th17 cells and IL-23 are important in the development of psoriasis. IL-23 stimulates the survival and the proliferation of immune cells [29]. In an individual with psoriasis, the production of IL-23 is increased by the dendritic cells and macrophages and is important for the development and maintenance of Th17 cells [30].

Ustekinumab is a humanized monoclonal antibody directed against p40, a subunit of IL-23 and IL-12. Although these two interleukins are produced by both dendritic cells and macrophages, they have distinct roles in the pathology. Therefore, ustekinumab specifically binds to IL-12 and IL-23 and inhibits their signal-transduction pathways that normally promote the differentiation of naïve T cells into Th1 and Th17, respectively [31]. More specifically, IL-12 promotes growth and differentiation of Th1 lymphocytes and cytotoxic T cells, whereas IL-23 stimulates survival and proliferation of Th17 cells. Finally, blocking IL-12 and IL-23 helps to reduce accordingly the number of Th1 and Th17 cells in the blood of patients with the disease. Indirectly, ustekinumab can decrease the production of IL-17, which is known to have the role of recruiting neutrophils in psoriatic lesions [32].

Apilimod is a small molecule that was developed from a novel triazine derivative and identified through a high-throughput IL-12 inhibitor screening [33]. Apilimod effectively suppresses synthesis of IL-12 and IL-23 in myeloid leukocytes, and oral administration of apilimod led to a suppression of the Th1 but not Th2 immune response in mice [33]. Recent research has established that apilimod not only suppresses the synthesis of IL-12, IL-23, and multiple downstream cytokines in the lesional skin, but also concomitantly increases synthesis of the anti-inflammatory cytokine IL-10 [34].

A recent review has described other signaling pathways targeted by new drugs currently under development for psoriasis [23]. Indeed, researchers are currently developing a new anti-IL-23 (SCH900222) that targets only the Il-23 (p19 subunit) and not the IL-12. This molecule is in Phase II of clinical trials.

As described previously, IL-17 is an important player in the survival of the disease. Several anti-IL-17 agents are currently in development or undergoing clinical trials. Brodalumab is a human monoclonal anti-IL-17 receptor A IgG2 antibody which inhibits the effects of IL-17A, IL-17F, and IL-17A/F [35], whereas secukinumab is a human monoclonal IgG1 monoclonal antibody to IL-17A [36].

Ixekizumab (LY2439821) is a promising new humanized IgG4 anti-IL-17 monoclonal antibody [37], which acts by blocking keratinocyte production of cytokines, beta-defensins, antimicrobial peptides (AMPs), and chemokines, thus multiple molecules that are found to be increased in psoriatic skin lesions [38]. Ixekizumab improves both pathologic skin features and clinical symptoms of moderate-to-severe chronic plaque psoriasis [37]. Ixekizumab is now undergoing Phase III clinical trials.

The “Toll-like receptor” (TLR) is a family of receptors that is expressed on the surface of various cell types, but particularly on the cells involved in innate immunity [39]. These receptors are able to recognize the pathogen-associated molecular patterns (PAMPs), such as lipopolysaccharides (LPS) or viral or bacterial DNA. If an agonist attaches to the TLR, it will cause the activation of different signaling pathways, which will cause the production of various inflammatory cytokines. TLRs and the signaling pathways that they activate are involved in the development of several diseases such as autoimmune diseases and cancer. The production of inflammatory mediators such as TNF-*α* and IL-6 is the consequence of the activation of TLRs. Consequently, the TLRs are very interesting targets for many diseases. In fact, it has been shown that when TLR7 and TLR9 are poorly regulated, there is a deregulation of the immune response that could eventually lead to the development of diseases such as psoriasis [39]. Therefore, TLR antagonists are under development to fight against psoriasis. The blocking of antibody receptors TLR7 and TLR9 is a new approach towards countering psoriasis. The company “Idera Pharmaceuticals” has developed an antagonist of TLR7 and TLR9 receptors (IMO3100). This antagonist blocks the activation of TLRs7, TLRs9, and MyD88 proteins, which normally activate signal transduction pathways leading to the production of inflammatory cytokines. This drug is currently in the preclinical phase [39].

## 4. Anti-T Cells Treatment

Alefacept is a fusion protein combining the Fc portion of the human IgG1 and LFA-3 (lymphocyte-function-associated antigen-3) located on antigen-presenting cells. This molecule has been developed specifically to modify the inflammatory process triggered with psoriasis [40]. This molecule specifically inhibits T-cell activation. The LFA-3 molecule is expressed on antigen-presenting cells. During the formation of the immunological synapse, it will bind to CD2 molecules expressed on mature T-cells and natural killer cells (NK). The binding of LFA-3 with CD2 molecule will generate important costimulatory signals in the process of naive T-cell activation in effector cells [41]. Psoriatic lesions contain mainly memory T-cells (CD45RO +) [42]. In addition, it is also important to mention that the CD2 molecule is overexpressed on lymphocytes (CD45RO +). alefacept blocks the interaction by the competitive inhibition of this interaction between LFA-3 on antigen-presenting cell and CD2 present on T-cells. The molecule binds to the T lymphocyte CD2 and prevents the formation of immunological synapse. This inhibition prevents the signal of costimulatory signal transduction between antigen-presenting cells and T lymphocytes. Therefore, this process prevents the activation and proliferation of T-cells and will then allow the induction of their apoptosis. alefacept also possesses another mechanism. It binds an overexpressed CD2 molecule on T-cells with the FcYIII receiver present on natural killer cells (NK) leading to the release of “granzyme” by NK cells and allowing the T-cell apoptosis [43]. In summary, on one hand, alefacept is capable of inhibiting proliferation and activation of memory T cells through inhibiting the binding of LFA-3 and CD2, and on the other hand, alefacept produces apoptosis of T cells through its role as mediator between the cell and the NK. In fact, this molecule produces good clinical results because it reduces the number of memory T cells and the number of CD4+ and CD8+ cells in the blood and it decreases the expression of INF-*α*, IL-8, and IL-23 in the psoriatic tissue [44]. However, alefacept is not available in Europe.

## 5. Small Molecule Inhibitors

### 5.1. Phosphodiesterase 4 Inhibitors

This phosphodiesterase (PDE) plays a key role in the degradation of adenosine monophosphate (AMP) in cells [45]. Inhibitors of phosphodiesterase will help prevent T-cell secretion of inflammatory cytokines such as TNF-*α* or IFN-*γ* and IL-2 from peripheral blood monocytes and T cells [46]. There are eight families of PDE, of which the PDE4 family is the most prevalent in immune cells and is expressed by keratinocytes [47]. Inhibition of PDE4 increases the intracellular concentration of cyclic adenosine monophosphate and subsequently reduces the production of proinflammatory cytokines [46]. Claveau et al.'s study showed that apremilast significantly reduces epidermal thickness and proliferation, decreases the histopathological appearance of psoriasis, and reduces expression of TNF-*α*, human leukocyte antigen-DR, and intercellular adhesion molecule-1 in lesioned skin [46]. Apremilast is a new oral therapeutic drug that inhibits phosphodiesterase 4.

### 5.2. Protein Kinase C Inhibitors

The protein kinase C (PKC) family is classified within a group of proteins bound to protein G [17] which contribute to several signal transduction cascades, playing an important role in the immune signaling cascade [35] and in the adaptive immune system. PKC are expressed in various types of cells that regulate immunological processes (development, differentiation, and activation of lymphocytes, macrophages, and dendritic cells) [48]. Indeed, sotrastaurin (AEB071) is an oral immunosuppressant that inhibits classical and novel protein kinase C isotypes [49] which are important for T-cell signaling [50] and for the production of INF-*γ* and IL-17 [51] which are key elements in psoriasis.

### 5.3. Mitogen-Activated Protein Kinase Inhibitors

Mitogen-activated protein kinase (MAPK) is very important in cell differentiation, proliferation, and inflammation. The importance of MAPK has been reported in many different inflammatory diseases [52]. The p-38 protein has awakened great interest as a potential molecular target for the treatment of psoriasis [17] because the p38-MAPK plays a key role in the biosynthesis of many inflammatory cytokines such as TNF-*α* [53], and the expression of p38-MAPK is overregulated in psoriasis lesions [54]. BMS582949 is a new selective p38 mitogen-activated protein kinase inhibitor.

### 5.4. Janus Kinase Inhibitors

The Janus kinases (JAK) are a family of cell-signaling molecules that are involved in the connection of several cytokine receptors to the signal-transducers and activators of transcription (STAT) pathways [55]. The activated STAT proteins control the expression of nuclear targets in genes and induce the transcription of proinflammatory genes [17]. JAK 1 and 2 have a role in the signaling of INF, whereas JAK 3 is involved in the signal transduction of IL-2, IL-7, IL-6, IL -15, and IL-21 [56]. The drug Tofacitinib (CP-690550) is designed to inhibit the isoforms 1 and 3 of the JAK kinase, and phase III clinical trials are already completed. The drug ASP015K inhibits JAK 3 and INCB28050 inhibits JAK 1 and JAK 2. There are other selective JAK3 inhibitors such as R348 and VX-509; however, they are still in the initial development phases [17].

### 5.5. Lipids

It has been reported that the gene for nitric oxide synthase (iNOS) increases the risk of psoriasis [57]. The INF-*γ* is an inducer of iNOS in macrophages and in dendritic cells. A higher production of oxidized lipids increases inflammation [58]. VB 201 is a treatment in Phase II clinical trials in psoriasis [59] and is designed to alleviate inflammation.

## 6. Nerve Growth Factor Inhibition

Research shows that there may be a link between emotional stress, the peripheral nervous system, and the onset of psoriatic lesions [60]. More precisely, during stress, sensory nerve fibers release neuropeptides in large quantities. Psoriatic lesional and nonlesional plaques contain a large amount of *nerve growth factor *(NGF) [35] which plays a role in keratinocyte proliferation, angiogenesis, T-cell activation, expression of adhesion molecules, and proliferation of cutaneous nerves [61]. K252a is a NGF receptor blocker, which has improved symptoms and histological features of psoriasis in a xenotransplant model [62]. CT327 is a topical treatment for an advanced stage of psoriasis and has been developed by Creabilis for the inhibition of TrkA kinase part of the NGF pathway.

## 7. New Therapeutic Approaches: Natural Treatments

For a long time, many unconventional treatments have been available for the treatment of psoriasis. Treatments involve vitamins, trace elements, and plant products. Polyphenols are organic molecules present in the plant kingdom. People are interested in antioxidant polyphenols and their effects on health. Some researchers believe that insufficient intake of antioxidants could contribute to the development of psoriasis [63]. Previously, it had been shown that the skin of people with psoriasis contained many radical hydroxyls [64] and a high level of nitric oxide [65], while it has been shown that natural polyphenols possess anti-inflammatory [66] and antiproliferative effects [67]. Research shows that a composition rich in polyphenols could downregulate the expression of calgranulins A and B genes (genes responsible for the production of inflammation) in a population of immortalized keratinocytes [68]. Studies were performed on the bark extract “*Picea mariana*” and showed that it has strong antioxidant and anti-inflammatory properties [69]. These studies have demonstrated that polyphenols extracted from the bark had antioxidant properties and were nontoxic to keratinocytes.

## 8. Research Perspectives

A recent study involving the role of insulin resistance in the development of psoriasis has shed new light on the subject [70]. Previous to this study, it has been shown that insulin had a role in maintaining the homeostasis of the skin [71]. In addition, other studies have highlighted the role of insulin in tissue repair [72]. It had been assumed that insulin resistance may have a potential role in the development and maintenance of psoriatic plaques or in signaling processes involved in psoriatic plaques. This recently published study [70] shows that IL-1*β* was readily detectable in the tissues and fluids surrounding the psoriatic plaques. In addition, IL-1*β* induces insulin resistance in the keratinocytes, which in turn alters their proliferation and differentiation. Older studies have reported that insulin was able to improve the differentiation of keratinocytes [71]. In fact, these results demonstrate that in healthy conditions, insulin is able to regulate cell proliferation and differentiation of keratinocytes with protein kinases, PI3-K protein and kinase B. In psoriasis, IL-1*β* is found in large quantities in the dermis. These large amounts of IL-1*β* lead to the activation of large amounts of p38-MAPK, which in turn induces insulin resistance by acting on insulin receptors, and then blocking cell differentiation. At the same time, IL-l*β* induces keratinocyte proliferation through the path of protein kinase JNK. In conclusion, these two pathways mediated by IL-1*β* represent the discovery of a new pathological mechanism that contributes to the development of psoriatic plaques. The results of this study have highlighted the role of insulin resistance in the development of psoriasis. These new advances can, therefore, help in developing new antipsoriatic treatments. 

## 9. Conclusion

The treatments available for psoriasis have increased rapidly in recent years; however, they are still incomplete. Although there are many drugs for different types of psoriasis, no drug can cure this pathology. In addition, many of them have serious side effects. Recent research has led to the development of new biological drugs that are produced through biotechnology which are effective for long-term. These biological treatments are an alternative to conventional treatments for moderate and severe psoriasis. Thanks to the discovery of new immunological factors and a better understanding of the functioning of psoriasis, researchers have turned their focus on immunological pathways and could gradually develop new biological drugs targeting pathways involved in the development of psoriasis. These biological drugs seem to be more effective and have fewer side effects than older, more conventional ones. However, their efficacy and long-term safety have not been fully tested, and furthermore, they are currently very expensive. The impact of this disease on the quality of life should encourage further research.

## Figures and Tables

**Table 1:** Drugs involved in the treatment of psoriasis.

Drug	Molecular target	Clinical trial phase	Administration route
Infliximab	TNF inhibitor	III	Intravenous
Etanercept	TNF inhibitor	Approved	Subcutaneous
Adalimumab	TNF inhibitor	Approved	Subcutaneous
Certolizumab Pegol	TNF inhibitor	III	Subcutaneous
Ustekinumab	IL-12/IL-23 inhibitor	Approved	Subcutaneous
Apilimod	IL-12/IL-23 inhibitor	II	Oral
Brodalumab	IL-23 inhibitor	III	Subcutaneous
Secukinumab	IL-17 inhibitor	III	Subcutaneous
Ixekizumab	IL-17 inhibitor	III	Subcutaneous
Alefacept	Anti T cell	Approved	Intravenous intramuscular
Apremilast	PDE 4 inhibitor	III	Oral
Sotrastaurin	PKC inhibitor	II	Oral
BMS-582949	P38 MAP inhibitor	II	Oral
Tofacitinib	Jak 3 inhibitor	III completed	Oral

Jak: janus kinase; p38 MAP: p38 mitogen-activated protein kinase; PKC: protein kinase C; PDE4: phosphodiesterase 4.
